# Supplementary material for: Being “resilient” and achieving “resilience”: From governmental discourses to the national research agenda in the contexts of food insecurity and cost of living
Source: PLoS One. 2025 Jan 3;20(1):e0316182. doi: 10.1371/journal.pone.0316182 (PMC11698476; doi:10.1371/journal.pone.0316182)
Supplement: S1 File — 50 first keywords (‘terms’) ranked by keyness score and classified in themes. (DOCX) [file pone.0316182.s001.docx]

# **Appendix 1**

Table 7 includes the first 50 keywords (‘terms’) for the GovCR and FundRC corpora using the .uk domain of the enTenTen20 as reference corpus. Terms are classified by theme and ranked according to keyness score. Raw frequency and keyness score are provided in-between brackets. Terms highlighted in grey in the ‘energy’ theme have also been included in another theme: ‘economy’ (‘energy security’, ‘energy market’), and ‘cost of living’ (‘energy bill’, ‘energy price’).

Table 7 – *50 first keywords (‘terms’) ranked by keyness score and classified in themes*

| **Theme** | **Government KW** | **Funders KW** |
| --- | --- | --- |
| Climate and environment | net zero (62/294,7) climate change (150/279,18) low carbon (34/156,49) climate action (31/147,85) electric vehicle (26/103,065) climate crisis (19/91,006) glasgow climate (18/86,268) environment agency (23/80,034) climate finance (15/72,057) green job (15/70,797) impact of climate (15/67,831) | net zero (72/336.06) climate change (147/268.79) coastal community (54/239.507) environmental sustainability (42/189.7) uk treescape (34/159.225) natural capital (33/153.208) |
| Food | food insecurity (71/331,44) food security (42/176,41) global food (21/93,027) food system (19/87,128) |  |
| Cost of living crisis | energy bill (59/247,45) cost of living (76/181,6) energy price (42/148,43) rising cost (28/74,94) |  |
| Economy | supply chain (79/323,42) energy security (38/177,85) monetary policy committee (30/141,85) inflation target (30/133,601) fuel duty (29/131,361) digital market (22/105,217) financial policy committee (21/100,48) credit union (30/91,175) employment allowance (18/85,507) price stability (26/82,862) price cap (17/78,728) energy market (18/70,056) competition enforcement (14/67,32) | supply chain (166/666.69) national capability (32/149.918) |
| People | mr speaker (31/134,67) vulnerable country (19/87,128) scottish government (19/85,668) |  |
| Energy | energy bill (59/247,45) energy security (38/177,85) energy price (42/148,43) offshore wind (36/141,34) energy system (29/109,986) energy market (18/70,056) |  |
| Research |  | project partner (226/1034,32), funding opportunity (183/816.293) proposed work (136/563.659) research organisation (150/458.345) project cost (100/398.904) uk research (91/374.484) text box (77/356.163) knowledge exchange (75/346.937) full economic cost (72/330.186) funding service (54/247.885) word count (59/245.032) principal investigator (64/244.44) responsible research (50/233.684) research hub (49/229.03) economic cost (75/224.265) research community (64/218.936) twinning research (45/210.415) digital twinning research (45/210.415) project lead (46/209.475) research area (68/203..93) project outcome (45/203.181) flexible fund (43/201.108) project co-lead (42/196.454) responsible innovation (42/196.454) assessment criterion (43/186.192) proposed research (45/175.958) eligible project (38/174.729) research office (38/173.214) research challenge (37/173.186) eligible project cost (37/173.186) letter of support (44/172.067) strategic theme (37/170.157) case for support (36/168.532) ukri funding service (36/168.532) career stage (36/165.584) grant funding (37/164.406) a4 page (43/157.616) start date (43/154.385) personal datum (49/152.843) host organisation (35/151.724) network plus (32/ 149.918) |
| Technology | tech sector (19/89,414) digital tech (16/76,794) | Digital twin (210/978.271) |
| Others | war of aggression (26/124,166) global challenge (26/121,994) gender equality (25/113,374) spring statement (22/105.22) charging infrastructure (18/86.268) covid-19 pandemic (17/81,531) humanitarian need (17/75,484) international partner (16/72.901) social care (40/71,016) policy framework (17/69,737) humanitarian assistance (16/65,685) |  |
