# Supplementary material for: Being “resilient” and achieving “resilience”: From governmental discourses to the national research agenda in the contexts of food insecurity and cost of living
Source: PLoS One. 2025 Jan 3;20(1):e0316182. doi: 10.1371/journal.pone.0316182 (PMC11698476; doi:10.1371/journal.pone.0316182)
Supplement: S2 File — Collocates of “resilience”; S2 File. Appendix 2 –Table 9. Collocates of “resilient”. (DOCX) [file pone.0316182.s002.docx]

# **Appendix 2**

Tables 8-9 show the WordSketch for “resilience” and “resilient” in the GovCR, FundRC, enTenTen20 and BNC corpora. Raw frequencies and typicality (strength) score of the collocates are indicated in brackets (first and second number respectively). Typicality score is calculated with LogDice (i.e., the higher the score is, the stronger the collocation is; the lowest, the higher probability for the words to collocate with other words). In the tables, words appear ordered per typicality score.

Table 8 – *Collocates of “resilience”*

| **Grammatical pattern** | **enTenTen20 (50,622 hits) (UK domain .uk subcorpus)** | **BNC (225 hits)** | **GOV (159 hits)** | **Funders (331 hits)** |
| --- | --- | --- | --- | --- |
| **Modifiers of “resilience”** E.g.: GOV: "to rebuild our fiscal resilience" | ***[16438/32.5]*** organisational (256/5.61) cyber (300/5.07) socio-ecological (18/4.88) wellbeing (65/4.59) preparedness (55/4.59) food-system (11/4.45) flood (234/4.38) teamwork (29/4.38) perseverance (16/4.33) confidence (125/4.3) | ***[76/33,8]*** playability (1/8,71) fortitude (1/8,69) steadfastness (1/8,65) sweetness (1/8,54) fault (4/8,3) hardness (1/8,22) stamina (1/8,1) indomitable (1/8,1) offers (1/8,04) unthinking (1/7,81) | ***[72/45,3]*** fiscal (5/10,2) financial (6/9,79) civilian (2/9,57) building (2/9,56) chain (2/9,5) long-term (3/9,42) climate (10/9,4) great (4/9,39) health (3/9,23) national (2/8,93) global (6/8,88) | ***[158/47,7]*** chain (15/11,2) supply (15/10,6) economic (14/10,4) building (6/9,93) treescape (5/9,79) climate (13/9,76) cultural (5/9,7) national (7/9,64) sustainability (4/9,51) increase (4/9,5) |
| **Nouns modified by “resilience”** E.g.: GOV: "actors on climate adaptation and resilience financing" | ***[8741/17.3]*** adaptability (33/5.52) resourcefulness (12/4.9) self-confidence (17/4.47) perseverance (16/4.13) Forum (443/3.85) tenacity (8/3.67) mindfulness (9/3.55) pathfinder (7/3.52) positivity (9/3.45) self-esteem (21/3.42) | ***[11/4,89]*** non-flexibility (1/11,4) malleability (1/11,2) stoicism (1/10) adaptability (1/9,21) quotient (1/8,96) defiance (1/8,08) courage (1/6,14) integrity (1/5,83) passion (1/5,36) present (1/4) | ***[25/15,7]*** programming (1/10), financing (1/9,95) program (1/9,91) Mission (1/9,91) mean (1/9,91) governance (1/9,79) package (2/9,53) building (1/9,53) collaboration (1/9,53) programme (3/9,43) | ***[68/20,5]*** energy (6/10,7) security (8/10,7) scenario (4/10,4) framework (4/10,4) theme (7/10) benefit (4/9,73) health (4/9,3) intervention (2/9,26) sustainability (2/8,82) programme (5/8,8) |
| **Verbs with “resilience” as object** E.g.: GOV: "decisions taken to rebuild our fiscal resilience" | ***[14940/29.5]*** strengthen (359/3.5) build (2853/3.44) bolster (36/2.85) enhance (416/2.85) improve (1352/2.78) conceptualize (8/2.59) underpin (26/2.59) boost (134/2.53) maximise (31/2.47) demonstrate (297/2.45) | ***[71/31,6]*** bristle (1/8,04) epitomise (1/7,57) understimate (2/6,85) celebrate (3/5,46) doubt (1/5,41) emphasise (2/5,2) display (3/4,97) respect (1/4,96) demonstrate (3/4,95) | ***[74/46,5]*** build (18/11,8) rebuild (6/11,1) strengthen (8/10,8) increase (12/10,6) show (3/9,81) test (2/9,64) enhance (2/9,38) boost (3/9,3) improve (3/9,09) undermine (1/8,54) | ***[110/33,2]*** increase (26/11,7) strengthen (11/11,3) build (19/11,3) improve (19/10,8) enhance (9/10,5) enable (5/9,47) explore (3/9,28) ensure (3/8,82) develop (3/8,05) deliver (2/7,56) |
| **“resilience” and/or …** E.g.: GOV: "This coordination has enhanced the strength and resilience of the UK's macroeconomic framework" | ***[19384/38.3]*** adaptability (174/6.84) wellbeing (602/6.84) resourcefulness (75/6.19) robustness (68/5.67) tenacity (75/5.66) self-esteem (127/5.51) confidence (545/5.51) determination (366/5.5) sustainability (395/5.43) preparedness (111/5.43) | ***[101/44,9]*** adaptability (4/9,48) firmness (2/8,66) tenacity (2/8,6) hardness (2/8,47) non-flexibility (1/8,31) savoir-faire (1/8,25) steadfastness (1/8,18) malleability (1/8,16) stoicism (1/8,16) quickness (1/8,15) | ***[56/35,2]*** strength (3/10,6) sustainability (3/10,4) adaptation (3/10,3) compassion (2/10,1) mitigation (2/10,1) kindness (2/10,1) security (4/9,75), action (2/9,41) agility (1/9,16) adaptability (1/9,16) | ***[117/35,4]*** security (29/12) sustainability (18/11,7) expansion (6/10,6) defence (5/10,1) productivity (4/9,71) emergency (3/9,67) health (5/9,5) adaptation (3/9,31) liveability (2/9,1) preparedness (2/8,96) |
| **“resilience” of…** E.g.: GOV: "programmes that build the resilience of vulnerable communities" | ***[3112/6.15]*** ecosystem (82/4.71) woodland (15/3.75) infrastructure (78/3.7) sector (83/3.33) reef (11/3.32) NHS (7/3.27) network (111/2.91) UK (27/2.61) supply (51/2.61) grid (11/2.59) | ***[39/17,3]*** warner (1/9,44) anarchism (1/9,38) fatherland (1/9,16) hardwood (1/8,89) mattress (1/8,76) positivism (1/8,55) savage (1/8,55) retailer (1/8,25) root (1/7,35) smile (1/6,91) | ***[15/9,43]*** economy (4/11,5) community (2/11,4) business (2/10,9) crop (1/10,9) leader (1/10,8) chain (1/10,8) population (1/10,7) **sector** (1/9,95) system (1/9,91) people (1/8,3) | ***[37/11,2]*** chain (6/11,9) UK (6/11,2) system (6/10,8) tree (2/10,7) grid (2/10,7) organisation (3/10,3) technology (2/9,46) |
| **“resilience” to…** E.g.: GOV: "Calling for a strengthening of global resilience to food insecurity" | ***[1552/3.07]*** radicalisation (15/7.78) Weather (9/6.86) shock (79/6.67) extremism (14/6.36) drought (47/6.17) flooding (50/6.01) hazard (35/5.62) Change (11/5.2) pest (11/5.17) disaster (43/4.73) | ***[13/5,78]*** throttle (1/11) abrasion (1/10,6) dip (1/10,4) wear (1/10) knock (1/9,87) recession (3/9,8) Monday (1/9,04) thought (1/7,5) treatment (1/5,9) fact (1/4,84) | ***[8/5,03]*** insecurity (1/11,3) vulnerability (1/11,7) drought (1/11,3) climate (1/11,3) threat (1/11,2) shock (1/10,9) crisis (1/10,9) change (1/9,95) | ***[36/10,9]*** hazard (6/12,2) change (9/12) risk (5/11,7) geohazard (3/11,3) pest (2/10,8) disease (2/10,6) |

Table 9 – *Collocates of “resilient”*

| **Grammatical pattern** | **enTenTen20 (22,790 hits) (UK domain .uk subcorpus)** | **BNC (212 hits)** | **GOV (90 hits)** | **Funders (152 hits)** |
| --- | --- | --- | --- | --- |
| **nouns modified by “resilient”** | ***[8907/39.1]*** Bars (58/5.03) lacquer (14/3.9) livelihood (27/3.83) mounting (15/3.79) cushioning (6/3.1) foam (47/3) GP (24/2.96) hardwood (11/2.85) Communities (22/2.83) NHS (6/2.73) | ***[78/36,8]*** self-belief (1/8,59) lillie (1/8,41) sinew (1/8,37) slither (1/8,34) cushioning (1/8,05) bounce (1/7,45) dunwoody (1/7,35) flooring (1/7,23) jockey (1/6,34) widow (1/5,94) | ***[42/46,7]*** workforce (2/9,95) community (3/9,76) chain (4/9,71) future (2/9,66) Asia (1/9,48) economy (4/9,43) NHS (1/9,41) crop (1/9,41) ecosystem (1/9,27) design (1/9,24) | ***[107/70,4]*** world (14/11,7) UK (9/10,8) society (5/10,1) theme (8/9,98) Hub (4/9,97) World (3/9,8) chain (10/9,76) infrastructure (6/9,76) farming (3/9,7) future (3/9,32) |
| **“resilient” and/or …** | ***[8299/36.4]*** resourceful (121/6.8) adaptable (132/6.53) sustainable (679/5.53) tenacious (27/5.53) liveable (13/5.15) determined (40/5.1) self-reliant (16/5.05), thriving (60/4.95) robust (238/4.9) confident (131/4.81) | ***[69/32,5]*** resilient (2/8,89) compressible (1/8,81) hard-headed (1/8,52) hard-wearing (1/8,46) robust (3/8,45) self-reliant (1/8,31) tempered (1/8,27) tenacious (1/8,23) bubbly (1/8,22) agile (1/8,13) | ***[43/47,8]*** sustainable (14/12) net (3/10,8) agile (2/10,4) secure (3/10,3) reliable (2/10,1) strong (2/9,93) investable (1/9,53) nature-based (1/9,53) diversified (1/9,53) friendly (1/9,53) | ***[89/58,5]*** secure (41/13,2) sustainable (19,11,7) digital (5/10,1) advanced (3/10) susceptible (2/9,49) flexible (2/9,37) efficient (2/9,28) climate-ready (1/8,5) cross-sectoral (1/8,5) rich (1/8,49) |
| **verbs before “resilient”** | ***[4650/20.4]*** prove (288/3.82) remain (399/2.44) build (17/2.24) become (478/1.35) develop (8/1.13) create (13/0.88) stay (39/0.8) | ***[56/26,4]*** prove (15/7,14) remain (2/3,1) look (1/1,65) seem (1/1,61) be (37/0,76) | ***[15/16,7]*** become (3/10,9) remain (1/9,44) be (11/8,41) | ***[7/4,61]*** represent (1/12) become (1/11,2) be (5/7,42) |
| **subjects of "be resilient"** | ***[1960/8.6]*** cyber (21/8) flood (32/6.94) sterling (6/5.97) livelihood (7/5.76) ecosystem (19/5.26) climate (56/5.19) infrastructure (32/4.41) economy (53/4.23) defence (8/4.23) organisation (31/3.86) | ***[18/8,49]*** ellipse (1/106) pollen (1/10,5) keyboard (1/9,95) chancellor (1/9,75) pursuit (1/9,75) custom (1/9,3) coach (1/9) nature (1/6,89), land (1/6,08) body (1/6,05) | ***[12/13,2]*** climate (5/13,2) chain (1/11,3) lot (1/11,3) finance (1/10,9) economy (1/10,8) system (1/10,8) market (1/10,5) business (1/10,4) | ***[13/8,55]*** advancement (3/12,5) region (2/12,1) UK (4/11,7) defence (1/11,1) sector (1/10,9) climate (1/10,8) system (1/10,5) |
| **“resilient” to …** | ***[1253/5.5]*** shock (83/6.76) rigour (6/6.44) flooding (54/6.14) downturn (18/6.08) scratch (7/5.81) moisture (14/5.35) stressor (8/5.06) disruption (14/4.56) impact (59/4.45) drought (13/4.34) | ***[9/4,25]*** adversity (1/11,3) handling (1/9,14) virus (1/8,85) depression (1/8,18) impact (1/7,92) error (1/7,75) infection (1/7,36) attack (1/6,83) use (1/5,01) | ***[5/5,56]*** climate (2/12,7) shock (2/12,2) impact (1/11) | ***[8/5,26]*** variability (4/13,4) pressure (1/11,8) shock (1/11,1) change (2/10,5) |
