# Supplementary material for: Being “resilient” and achieving “resilience”: From governmental discourses to the national research agenda in the contexts of food insecurity and cost of living
Source: PLoS One. 2025 Jan 3;20(1):e0316182. doi: 10.1371/journal.pone.0316182 (PMC11698476; doi:10.1371/journal.pone.0316182)
Supplement: Attachment — Submitted filename: Response to reviewers.docx [file pone.0316182.s003.docx]

Dear Dr Tomassi,

Thank you for providing us with the reviews and offering us the opportunity to revise the manuscript “Being “resilient” and achieving “resilience”: from governmental discourses to the national research agenda in the contexts of food insecurity and cost of living” to PLOS ONE. We strongly believe that we have addressed all the comments raised by the reviewers in the most concise and coherent manner. For clarity, responses to reviewers’ comments are provided in blue below each comment. We have included the data availability statement in the MS as requested as per PLOS ONE guidelines. We are currently working with the university to make part of the data available in a repository (link not available yet).

We would also like to thank you, and the reviewers, for the time and the comments provided.

Kind regards,

The authors

***

## **Editor**

Reviewer 1 suggests that your historical overview of 'resilience' lacks a discussion on organizational resilience and resilience engineering. These areas, particularly as developed by scholars like Erik Hollnagel, could significantly deepen the theoretical grounding of your paper. Incorporating these perspectives could provide a more comprehensive view of how resilience functions not just at individual or community levels but also within complex socio-technical systems.

A brief discussion of Engineering Resilience has now been included –please see response to Reviewer 1, comment 1, provided below.

Reviewer 2, in particular, mentions the misalignment between the title, introduction, and the study's objectives. Clarifying how each section of your manuscript contributes to your stated aims will help strengthen your argument's logical flow. Moreover, expanding on your focus group methodology—such as the criteria for participant selection and the rationale behind the chosen questions—will enhance the validity of your findings.

The misalignment has now been amended and more detail on the focus group has been provided in the methodology –please see response to Reviewer 2, comments 1, 3, 4 and 5.

The concern about selection bias, as raised by Reviewer 1, is crucial. Your focus on specific elements of resilience within governmental discourse might benefit from a broader lens. Consider how you might include additional data sources or expand your analytical framework to mitigate this bias. This could involve examining a wider range of documents or incorporating more diverse viewpoints from different sectors or geographical areas within the UK.

Please see response to Reviewer 1, comment 3 on this matter. As we explain there, we do not understand that building a specialised corpus with governmental texts and another one with funding calls texts to answer a research question (RQ 2) about governmental discourses and research funding calls specifically incurs in selection bias –building up specialised corpora is common practice in studies applying corpus linguistics to discourse analysis, as this one. We completely agree that the data would be biased if we were using these small specialised corpora to make general claims of language use in British English beyond the registers considered, but this is not the case. Limitations of extrapolation of findings derived from data restrictions are included in the new Section 6.2 ‘Limitations and future research directions’ – please see answer to Reviewer 2, comment 8.

There is a noted need for justifying the geographical focus on the UK more robustly. Discuss why the UK presents a unique or particularly informative case for studying resilience in the context of food insecurity and cost of living. This could involve more explicitly linking your findings to current debates or policy initiatives within the UK.

The focus on the UK context has now been justified –please see response to Review 2, comment 2.

Ensure that the term 'resilience' is consistently defined and used throughout the paper. As resilience is a multi-dimensional concept, a clear and operationalized definition at the beginning of your paper will help maintain focus and prevent the ambiguity noted by Reviewer 1.

The concept of ‘resilience’ has now been operationalized —please see response to Reviewer 1, comment 2.

## **Reviewer 1**

**1.** **[Expansion of theoretical framework to incorporate the concepts of ‘engineering resilience’ and ‘organizational resilience’]:** “The authors, in their brief and unfortunately not exhaustive historical overview, cite seminal works by McGreavy, Frydenberg, Woods, and Holling, tracing the evolution of the term resilience from its original Latin meaning through its usage in cognitive and ecological sciences. However, they omit an important semantic facet recognized by a growing academic community, which could have been uncovered and analyzed by further investigating Woods' work: organizational resilience or resilience in the sense of Resilience Engineering. In the context of this study, this perspective would have offered an integrated and fundamental viewpoint on the subject. This vision, developed by experts like Erik Hollnagel, focuses on the ability of socio-technical systems to maintain safe and continuous operations in uncertain and changing contexts. Hollnagel defines resilience as the capacity of a system, whether technical, human, or organizational, to adapt, absorb, and rapidly recover from disruptions or changes. This vision emphasizes four main capabilities: responding, monitoring, anticipating, and learning. These capabilities apply not only to individuals but also to collectives and organizations, demonstrating how resilience can be a property of entire socio-technical systems. The evolution of the concept of resilience as described by Resilience Engineering is a direct response to the inadequacy of traditional safety and security concepts, which no longer meet the needs of modern complex socio-technical systems. These systems, which include critical infrastructures, complex organizations, and global networks, require a more dynamic and adaptive approach to manage uncertainty and disruptions. The concept of complexity and complex adaptive systems (CAS) offers a crucial perspective on resilience, highlighting how it emerges from both internal interactions and external relationships. This approach not only challenges the researchers' partial and potentially distorted interpretations but also demonstrates that resilience is an intrinsically dynamic and multifactorial quality. The coexistence of internal and external forces makes the described framework more coherent and diminishes the strength of their critiques, underscoring the importance of considering resilience as an emergent and adaptive property in modern socio-technical systems. To claim that resilience should not be attributed to collective agents is reductive. Communities, organizations, and entire nations must be resilient to face global challenges. Individual psychological resilience is just one component; collective resilience is crucial to ensure that complex systems can adapt and thrive. Critical analyses that ignore this collective dimension risk promoting a limited and unrepresentative view of reality. Consequently, the presence of resilience in the political and research agendas is not only justified but necessary. Global challenges such as climate change, economic crises, and pandemics require a systemic approach. In this context, resilience becomes an essential framework for developing policies and practices that enable social, economic, and technical systems to confront and overcome disruptions.”

“Ultimately, it seems that the authors have (re-)discovered an additional facet of the construct "resilience" according to resilience engineering. Resilience engineering provides a framework that recognizes the complexity and adaptive nature of modern systems. Instead of viewing resilience as a simple individual recovery capacity, resilience engineering considers it an emergent property of complex systems, where dynamic interactions and interdependencies between internal and external components play a crucial role. Resilience engineering aligns with constructivist approaches, which see knowledge and reality as socially constructed through continuous interaction between human and non-human actors. This approach acknowledges that resilience is not a static characteristic but a continuous process of adaptation and transformation. Actor Network Theory (ANT) further contributes to this vision, emphasizing that agency is distributed among networks of both human and non-human actors. According to ANT, resilience emerges from the relationships among these actors, including individuals, technologies, infrastructures, and institutions. This perspective broadens the concept of resilience beyond the individual level, encompassing the entire network of interconnected actors. When reinterpreted through the lens of resilience engineering and ANT, research themes and the political agenda become more coherent and relevant. Here's how:

- Critical Infrastructure Systems: Research on the resilience of critical infrastructures, such as energy, water, and transportation, becomes fundamental. Instead of focusing solely on incident prevention, resilience engineering promotes adaptability and recovery capabilities through dynamic design and management.
- Environmental and Climate Policies: Environmental policies aimed at combating climate change gain a new dimension. Resilience engineering encourages the adoption of sustainable and adaptive practices that allow communities and ecosystems to effectively deal with climatic disruptions.
- Socio-Economic Development: Economic and social resilience is viewed through the prism of interactions among various actors. Policies promoting socio-economic resilience must consider the interdependencies between communities, institutions, and technologies, fostering networks of support and collective innovation.
- Public Health: In public health, resilience engineering suggests approaches that strengthen health systems through continuous learning and adaptation, recognizing the crucial role of networks of involved actors, from health institutions to patients, from medical technologies to health policies.”

Thank you very much for directing us to Resilience Engineering and providing such a detailed description, this has been very helpful. Resilience Engineering has now been included in the theoretical discussion provided at Section 3.1 (please see paragraph copied below), and has been adopted to operationalize the concept for this study (please see response to comment 2, below).

*A relatively recent approach to the conceptualisation of resilience is the one proposed by Resilience Engineering (e.g. Dekker et al. 2008; Hollnagel, 2014). Following Resilience Engineering, a system (an organisation, group or individual) is resilient if it has the capacity to effectively “adjust its functioning prior to, during, or following changes and disturbances, so that it can continue to perform as required after a disruption or a major mishap, and in the presence of continuous stresses” (Dekker et al. 2008: 53). Adjustment to unexpected changes to continue functioning in the new conditions is associated with the ability to respond to changes, but also to monitor, learn and anticipate future challenges (Holling, 2014). In contrast to the early uses, which would associate resilience with the recovery of a previous state or the capacity to withstand stress, Resilience Engineering highlights resilience as developing capacities for adjustment to continuous environmental changes.*

**2. [Theoretical framework: Operationalization of the concept ‘resilience’]:** “The article focuses on a concept known for its multidimensional nature and varying interpretations depending on the context. The lack of a clear and operationalized definition of "resilience" uniformly applied throughout the analysis can lead to ambiguous or less rigorous conclusions.”

Thank you for pointing out that providing a definition to operationalize the concept as adopted in this paper could enrich the study. As commented above, the conceptualisation of Resilience Engineering has now been adopted as suggested (please see below, included in section 3.1 as closing).

*In this study, we follow the more holistic definition provided by Resilience Engineering, which conceptualises resilience as adaptative property of individuals and social groups that emerges through constant interaction with the environment or societal context.*

**3. [Data]:** “I believe the authors have fallen into a selection bias that may compromise the validity of their findings. ”
“The authors chose to use governmental discourses and funding calls as their primary sources. Limiting the analysis to these documents may have reinforced the selection bias, inadvertently consolidating a frame where the discourse of safety and security, and thus the collective behavior of social, technological, and research systems (including family management as an organization), simply does not exist. This greatly limits the various facets and contexts in which the concept of "resilience" is applied.”

We understand the concerns expressed in this comment. The data selection would fall into selection bias if this study attempted to give account of the current social understanding of ‘resilience’ and only considered the two registers included in the datasets gathered (governmental and funding bodies discourses). However, please note that the selection of governmental and funding discourses to construct the two datasets or specialized corpora follows from the objectives of this study as defined in the research questions, specifically research question 3 –i.e. to observe any differences / similarities between the use of ‘resilience’ by governmental discourses and those of the funding bodies. It is thus to be expected that the findings obtained from this analysis will be limited to these uses observed in the datasets considered and will not reflect other themes that may be prevalent in other areas and as such should not be extrapolated. This has now been more explicitly stated in the methodology as copied below, and the limitations have been acknowledged in the concluding discussion (please see “Section 6.2 Limitations and future research directions” added at the concluding discussion –relevant paragraph copied below).

**[Methodology]** *We adopt a corpus-based approach to examine the use of ‘resilience’ in governmental discourses and UK national research funding calls and identify any shared patterns of meaning between these two domains (research question 3). Two specialised corpora were built for the purpose of this study …*

*Specialised corpora aim at being representative of a particular register or linguistic variety, for this study the UK governmental and academic communications around resilience. Therefore, uses of ‘resilience’ observed in the specialised corpora (GovRC, FundRC) cannot be extrapolated to the general British English. Uses of ‘resilience’ in general British English (research question 2) were examined in two reference corpora: the British National Corpus (BNC), and the English Web enTenTen20 (enTenTen20), both of them available in Sketch Engine.*

**[Section 6.2]** *While this study specifically focused on examining uses of resilience in governmental discourses and funding calls to identify possible correspondences between these two domains, restricting the data to these registers has inevitably limited the uses of ‘resilience’ to those that appear in these texts. Examining uses of ‘resilience’ in health communication discourses or news media would provide a more encompassing overview of the different conceptualisations and areas of application of the concept, illustrating how specific disciplines adopt it (e.g. health sciences vis-à-vis politics) and how it is communicated to the public.*

**4. [Focus groups]:** “Additionally, the number of focus groups conducted and the demographic composition of participants are not specified. If the groups are not representative of the general population or specific subgroups, the results may not be generalizable.”

Please note that the number of focus groups and participants was specified (see words in bold in the paragraph below); a note on demographics has also been included (underlined below). The limitations regarding extrapolation have been acknowledged at the concluding discussion (Section 6.2, copied below). Please note, that the limitation about extrapolation is part of focus group-based qualitative research.

***Two one-hour focus groups*** *were held in December 2023 and were facilitated by DC via MS Teams.* ***The focus groups comprised six participants overall****.* *Participants were recruited by an email invitation advertising the study to people who had previously contributed towards patient and public involvement (PPI) activities for the University of Southampton. Since we were interested in collecting lay perspectives on resilience, none of the participants recruited reported professional or academic expertise on the subject during the recruitment process. Other demographic information was not collected from participants for this study.*

**[Section 6.2]** *Findings of this study are restricted to the UK context; populations with different cultural, sociopolitical and economic traditions may show different understandings and applications of the concept. Importantly, the limited number of focus group participants makes it difficult, if not impossible, to extrapolate findings to the general population. Lack of demographic information does not make it possible to examine whether understandings of resilience vary across different social groups—e.g. some focus group participants observed that different age groups show different orientations towards the concept, with elder populations conceptualising resilience in relation to material conditions while young populations understand it in relation to psychological wellbeing and the individual ability to seek support. Follow-up studies on lay understandings of the concept should complement focus groups with other public involvement methods which allow for more efficient data gathering and the possibility to scale-up findings, such as questionnaires including both closed and open questions (see, for example, McClaughlin et al., 2023 or Vilar-Lluch et al., 2024 for mixed-methods studies combing corpus linguistics with representative surveys applied to public health guidance reception).*

**5. [Methods]: “**While the use of corpus-based analysis is a solid approach for studying linguistic usage in large datasets, the validity of the results heavily depends on the tools and analysis procedures employed. Without details on the criteria for selecting keywords, statistical significance metrics, and normalization procedures, there might be concerns about the accuracy of the analysis. Relying uncritically on standard linguistic analysis software solutions only heightens my doubts. The choice of collocation analysis seems appropriate, less so the relative frequency analysis. Sometimes, as you surely know, the presence of hapax legomena can be more explanatory than frequent words for interpreting text semantically. Moreover, an analysis of the lexical richness of both the documents and the linguistic capacity of the target referent might be more appropriate in this context.”

Thank you for giving us the opportunity to clarify potential misunderstandings and for commenting on the lack of development of keywords –keywords were mentioned in the methodology but were not reported in the study. This has now been amended as noted below.

Keywords - Methodology section:

*Keywords are words that occur “with unusual frequency” in the corpus under study “by comparison with a reference corpus of some kind” (Scott, 1997: 236), thus making it possible to identify the most salient themes of our corpus. Sketch Engine adopts the metrics of ‘simple maths’ to calculate ‘keyness’ score and retrieve keywords. Simple maths involves the comparison of the normalised (relative) frequencies of the words in the focus corpus with those of the reference corpus and makes it possible to compare between corpora of different sizes 5 (see Gabrielatos, 2018 for keyness metrics). Keywords may be single-token items or multiword expressions (‘terms’). For this analysis, we considered the first 100 terms (ranked by keyness score) for both the GovRC and FundRC, and classified them into themes defined by checking their use in context with the concordance tool. The concordance tool retrieves strings of text displaying the expression of interest in context, making it possible to examine how it is used, identify linguistic patterns, and infer evaluations (Partington & Marchi, 2015: 216). We acknowledge that the 100 terms cut-off is arbitrary, but it allowed us to identify the most salient topics for each dataset and reach saturation –i.e. the point in which new themes do not emerge. Appendix 1 provides an overview of the first 50 keywords (‘terms’) of each corpus classified by theme. Keywords were retrieved using the .uk domain sub-corpus of the enTenTen20.*

**[Please see Appendix 1 for the table referred in this paragraph].**

Please note that calculation for retrieving KW in SketchEngine was specified in Footnote 3, now Footnote 5, including normalisation (please see below). Smoothing parameter used for the analysis has been now included (underlined).

**[Footnote 5]** *The formula to calculate keyness score reads: (fpm_focus_ + N) / (fpm_ref_ + N) —“fpm_focus_” and “fpm_ref_” are the normalised frequencies (per million) of the focus and reference corpora respectively, and “N” the smoothing parameter. By default, N = 1; N > 1 (e.g. 10, 100…) allows us to focus on higher-frequency words, and N < 1 (e.g., 0.1, 0.01), allows us to focus on lower-frequency words (see* [*https://www.sketchengine.eu/documentation/*](https://www.sketchengine.eu/documentation/)*). For this analysis, N=1.*

Keywords – Analysis section

The overview of the specialised corpora provided at Section 4.1 now includes some insights from the keyword analysis to complement the observations of the relative frequencies –please see below.

*Examining the keywords provides a more refined overview of the ‘aboutness’ of each corpus (Appendix 1, Table 8). ‘Economy’ (e.g. supply chain, inflation target) and ‘climate and environment’ (e.g. net zero, climate change) feature as the most recurrent themes in the governmental discourses (GovRC), together with ‘food’ (e.g. food insecurity, food security) and ‘cost of living crisis’ (e.g. energy bill, cost of living) themes. The topics of food insecurity and cost-of-living crisis were intentionally included in the query to build the specialised corpora, and references to these themes should thus be expected. The recurrent references to the environment and economy are remarkable, suggesting that references to resilience tend to appear in relation to these matters in governmental discourses. The double-coding of some keywords as providing references to energy and as being used in relation to economy (energy security, energy market) or cost-of-living crisis (energy bill, energy price) evidences the energy crisis as one of the main shocks that need resilience, both from the individual or household level, and from the national economy (see Appendix 1, Table 8).*

*In consonance with the recurrent use of terminology related to funding applications revealed by the wordlists (Table 3), ‘research’ is the most prominent theme identified for the keywords of the funders’ corpus (see Appendix 1, Table 8)— 41 out of the first 50 ‘terms’ of the FundRC refer to research-related matters, such as types of documentation and individual roles involved in applications. Remarkably, if register-specific jargon is excluded, the most salient theme is ‘climate and environment’ (e.g. net zero, climate change) and, in a much lesser extent, economy (supply chain, national capability), which reflects the themes identified in the governmental discourses.*

Calculation of relative frequencies

Please note that base for normalisation to calculate the relative frequencies of collocates referring to social actors is indicated under Table 6, where these RF are included (i.e. “*Relative frequencies (RF) normalised per 1000*.”) and at footnote 9, now 12, when discussing occurrences of collocates (i.e. “*RF calculated with 1000 base*.”)

Collocates – Methodology section

The description of collocates in the methodology section has been expanded as noted below (underlined) —now it specifies the grammatical patterns considered and rationale for doing so:

*Collocates were extracted with the WordSketch tool. WordSketch organises the collocates into grammatical categories, thus providing an insight into how the collocates are related to the node term (‘resilience’, ‘resilient’) and function in context. In particular, we were interested in those grammatical patterns that allowed us to identify: types of resilience (collocates pre-modifying ‘resilience’); entities that may be attributed resilience (noun collocates modified by ‘resilience’/‘resilient’, subjects of the phrase ‘be resilient’, or collocates for the prepositional phrase ‘resilience of’); entities that may be perceived as shocks in need of resilience (collocates for the prepositional phrases ‘resilient to’, ‘resilience to’); actions associated with resilience (verbs collocating with ‘resilience’ as object); and qualities or entities commonly associated with resilience (phrases ‘resilience and/or’, ‘resilient and/or’)-. Appendix 2 provides an overview of the collocates for ‘resilience’ and ‘resilient’ classified by grammatical pattern. Sketch Engine measures collocation strength (how typical a co-occurrence is) with logDice (Rychlý, 2008: 9). The maximum theoretical value is 14 (absolute co-occurrence of two words), and negative values mean that the co-occurrence of the terms is not statistically significant. LogDice is not affected by corpus size, thus allowing us to compare different corpora.*

Distribution:

As per Reviewer’s 2 comment 7, a brief note on dispersion has been included (Section 4.1). A note on how results can be (highly) dependent on the measures adopted has been included as a footnote—please see below:

*In comparison, the distribution^10^ of the terms in our corpora appears relatively even—Juilland’s D^11^ for ‘resilience’ and ‘resilient’ in the GovRC is 0.8881 and 0.8528 respectively, and in the FundRC is 0.8625 and 0.7818 respectively. The moderately even distributions of our corpora can partly be explained by register-specificity (each corpus exclusively contains texts of one register) and by the search terms used for corpus building.*

**[Footnote 10]** *Dispersion calculated with the software #LancsBox 6.0 (Brezina et al. 2021).*

**[Footnote 11]** *Juilland’s D falls between 0 (very uneven distribution) and 1 (very even). Gries’ DP-norm (normalised deviations proportions, sometimes preferred in corpus linguistics, see Gries 2020), which falls between 0 (very even distribution) and 1 (very uneven), provides moderately even distributions, albeit comparatively less— DP-norm for ‘resilience’ and ‘resilient’ in the GovRC is 0.4914 and 0.5519 respectively, and in the FundRC is 0.3885 and 0.4387 respectively.*

Please note, however, that this study does not consider the genre differences between the datasets considered beyond those differences in using ‘resilience’ (now explicitly stated, please see sentence copied below, term in bold) – examining lexical richness would provide a good examination of genre characteristics, but falls beyond the objectives of this study as defined in the research questions (provided at the introduction). In the same manner, we do not consider reception of the documents included in the datasets (i.e. how the British public understood the political discourses examined or how particular academics understood the funding calls); as mentioned, the analysis exclusively focuses on the use of the concept ‘resilience’. Therefore, “the linguistic capacity of the target referent” has not been examined, since this would require a completely different study and methods of analysis. We understand that the term “construal” (previously used instead of “use” in the sentence below) also connotes “understanding” besides “representation”; the term has now been removed to avoid misunderstandings.

*We adopt a corpus-based approach to examine the* ***use*** *of ‘resilience’ in governmental discourses and UK national research funding calls.*

## **Reviewer 2**

**1.** There is a shift in the objective between the title and the introduction and research questions. The title and the query used in the methodology focus on the topic of food insecurity and cost of living, whereas the introduction does not address this topic. Please clarify this point by introducing the topic of food insecurity and cost of living in the introduction to align it with the title and methodology.

Thank you for highlighting this inconsistency. The research questions have been rephrased to reflect better the focus of the study as copied below. A brief discussion of food insecurity and cost-of-living crisis in the UK context has now been included in the Introduction (please see response to comment 2 below).

*In this paper, we operationalise ideas from Critical Policy Discourse Analysis (Mulderrig, Montesano Montessori and Farrelly, 2019) with methods from corpus assisted discourse analysis to examine institutional and lay understandings of ‘resilience’ within the British public by addressing these research questions:*

*(1) What are the academic and political origins of the concept ‘resilience’?*

*(2) Do general British English corpora (large collections of digitised texts representative of a language) show any changes in the use of ‘resilience’ over time?*

*(3) Can we observe any specific patterns of use of the term ‘resilience’ for governmental communications and funder calls as compared to general use, particularly in relation to the contexts of cost-of-living crisis and food insecurity?*

*(4) Do members of the public associate ‘resilience’ with the contexts of cost-of-living crisis and food insecurity? What meanings do they orient to otherwise?*

**2.** The study focuses on a British context. It is important to introduce and justify the relevance of this geographical focus. Explain why the British scenario is significant for the study and eventually address limitations for this focus.

Thank you for this comment. The British context is now introduced in the Introduction section as the focus of the study and a rationale is provided as copied below:

*In the context of the United Kingdom (UK), the pervasiveness of the concept has extended into the publicly funded research agenda.*

*We aim to shed a light on this dynamic by examining the meaning of ‘resilience’ in the contexts of household food insecurity and cost-of-living crisis in the UK by analysing its use in governmental discourse, research funding calls and the social community in focus groups. The recovery from the global Covid-19 pandemic dramatically intensified the cost of living in the UK (ONS, 2024). Economic pressures from the global recovery of the Covid-19 pandemic and the disruption of supply chains caused by the Russian-Ukrainian war have been reported globally. In the UK context, the aftermath has been particularly severe. In March 2023, the rate of increase in food prices (19.1%) was reported to be the highest since the economic stagnation of 1977, and by May 2024 it had risen by 30.6% since May 2021 (Francis-Devine et al., 2024). The severity of food insecurity among deprived communities has risen the alarm of widening health inequalities across the country (Food Foundation, 2024a).*

Limitations derived from focusing on the British context exclusively have also been accounted for at the Limitations section now included as part of the concluding discussion (please see response to comment 8 below).

**3.** The focus group target appears to be quite generic. Are the participants experts in food insecurity and/or cost of living? How can their expertise in the topic of resilience be assessed? Provide clear criteria or justification for the selection of focus group participants to ensure their relevance to the study.

The following explanation has been provided when describing the focus group (section 2.3):

*Participants were recruited by an email invitation advertising the study to people who had previously contributed towards patient and public involvement (PPI) activities for the University of Southampton. Since we were interested in collecting lay perspectives on resilience, none of the participants recruited reported professional or academic expertise on the subject during the recruitment process. Other demographic information was not collected from participants for this study.*

Difficulty to extrapolate results of the focus group analysis to the general population has been accounted for at the Limitations section now included as part of the concluding discussion (please see response to comment 8 below).

**4.** The questions addressed to the focus groups are very generic and not specifically focused on food, which makes the analysis not comparable to the Corpus-based Discourse Analysis and Critical Policy Discourse Analysis. Justify the choice of questions and explain how they align with the objectives of the study.

The following explanation has been added to make the rationale for question design clear—while the study was specifically focused on the uses of ‘resilience’ in the contexts of cost-of-living and food insecurity, we were interested to see whether this was an association that focus groups participants would make in the first place:

*Group conversations were directed using the open questions displayed in Table 2. Questions were purposefully designed to be as open as possible in order not to precondition responses and obtain participant’s genuine views on the meaning of the concept ‘resilience’. […] Absence of questions with explicit references to the topics of cost-of-living crisis and food insecurity allowed us to determine whether participants would associate resilience to these societal crises in the first place, as the UK societal context of the time would have suggested. Specifically, questions around context of use of the term and examples (Q3, Q5 and Q6, Table 2) were designed to act as prompts to elicit any associations between resilience and societal and economic conditions.*

**5.** The term “example prompt” used in Table 2 is unclear and there are no references to this in the article. Clarify what is meant by “example prompt”.

Thank you for pointing this out. The following clarification has been added over Table 2:

*Follow-up questions were used to elicit more information when required (e.g. if participants did not seem to understand the question as it had been originally formulated—see ‘example prompts’, Table 2).*

**6.** The topic of COVID-19 emerged in the focus group discussions, which is certainly linked with resilience and food insecurity. There have been multiple research studies addressing how the concept of resilience has adapted to the pandemic and the topic of food insecurity and food supply chains. Add a section in the background discussing the impact of COVID-19 on resilience and food insecurity, citing relevant studies to provide a comprehensive context.

A section on resilience and Covid-19, specifically focusing on the UK and food insecurity contexts has now been added (Section 3.4 “Resilience and Covid-19”, copied below).

*3.4 Resilience and Covid-19*

*By May 2021, a year into the global Covid-19 pandemic, global food prices had increased by more than a 40% since May 2020, while food prices in local markets were conditioned by a cluster of factors including supply and demand, transportation costs, governmental policies and income levels (IMF, 2021: 58). The global pandemic led to considerations of how societies can be more resilient to crises (e.g. Barker & Russell 2020; Béné, 2020; Klassen & Murphy, 2020; Sanderson et al. 2021). Particular attention has been paid to the resilience of rural communities, understood to experience higher demographic pressures and limited industrial diversity and policy resources which may exacerbate the impact of the crisis (McIntyre & Roy, 2023), and to the context of food insecurity. Food insecurity is regarded as a marker of wealth distribution in a society, economic redistribution and political accountability (Klassen & Murphy, 2020). Thus, the sharp increase in food insecurity during the pandemic attested systemic weaknesses in food, social and economic systems, and in many contexts evidenced an unequal distribution of societal risk, with some populations being significantly more vulnerable than others, such as in the UK (Glass et al. 2024; Sanderson et al. 2021: 783). In the UK context, the global health crisis is sometimes regarded as part of a broader ‘permacrisis’ or ‘polycrisis’, i.e. “a state of ongoing disruption to political, economic and social life” which in the UK includes Brexit, years of austerity policies and the crisis of the National Health System (NHS), which merge with the global health and financial crises, the war in Ukraine and increase in energy prices, and climate change (Glass et al., 2024: 50). Against this backdrop, the pandemic has widened health inequalities, has evidenced the weakness of relying on food charities and the voluntary sector as primary response to poverty in the UK context (Barker & Russell 2020: 868; Power et al., 2023), and has revealed general global lack of preparation for responses balancing the trade-off between health and economy (Béné, 2020: 817).*

*Resilience to crisis, or the ability to absorb the shock, reorganise and recover while undergoing change (Klassen & Murphy 2020), has been attributed both to the severity of the shock and the measures adopted (Béné, 2020: 817). Studies on the impact of, and responses to, the Covid-19 pandemic have highlighted the importance of market diversification and promotion of local food systems (e.g. food cooperatives, community supported agriculture) and businesses to empower local communities, diminish over-reliance on imported food and increase the resilience of the food sector (Jones et al., 2022; McIntyre & Roy, 2023; Sanderson et al. 2021: 790), and the need for governmental support (Power et al. 2023), including recommendations to strengthen “social safety nets and income floors” such wealth redistribution measures or a Universal Basic Income (Klassen and Murphy, 2020).*

**7.** The authors have only analyzed the absolute frequency of terms. It might be interesting to also analyze term frequency-inverse document frequency (tf-idf) to identify if some specific documents or topics are more relevant. Highlighting the importance of terms in specific documents relative to their occurrence in the entire dataset can provide deeper insights into the data.

Thank you for this comment. Distribution of ‘resilience’ and ‘resilient’ has now been included in Section 4.1 as copied below. An overview of salient themes emerging from keyword analysis of the two specialised corpora (GovRC and Fund RC) has also been included in Section 4.1, a summary table is provided as Appendix 1.

Distribution discussion:

*In comparison, the distribution10 of the terms in our corpora appears relatively even—Juilland’s D11 for ‘resilience’ and ‘resilient’ in the GovRC is 0.8881 and 0.8528 respectively, and in the FundRC is 0.8625 and 0.7818 respectively. The moderately even distributions of our corpora can partly be explained by register-specificity (each corpus exclusively contains texts of one register) and by the search terms used for corpus building.*

**[Footnote 10]** *Dispersion calculated with the software #LancsBox 6.0 (Brezina et al. 2021).*

**[Footnote 11]** *Juilland’s D falls between 0 (very uneven distribution) and 1 (very even). Gries’ DP-norm (normalised deviations proportions, sometimes preferred in corpus linguistics, see Gries 2020), which falls between 0 (very even distribution) and 1 (very uneven), provides moderately even distributions, albeit comparatively less— DP-norm for ‘resilience’ and ‘resilient’ in the GovRC is 0.4914 and 0.5519 respectively, and in the FundRC is 0.3885 and 0.4387 respectively.*

Keywords discussion:

*Examining the keywords provides a more refined overview of the ‘aboutness’ of each corpus (Appendix 1, Table 7). ‘Economy’ (e.g. supply chain, inflation target) and ‘climate and environment’ (e.g. net zero, climate change) feature as the most recurrent themes in the governmental discourses (GovRC), together with ‘food’ (e.g. food insecurity, food security) and ‘cost of living crisis’ (e.g. energy bill, cost of living) themes. The topics of food insecurity and cost-of-living crisis were intentionally included in the query to build the specialised corpora, and references to these themes should thus be expected. The recurrent references to the environment and economy are remarkable, suggesting that references to resilience tend to appear in relation to these matters in governmental discourses. The double-coding of some keywords as providing references to energy and as being used in relation to economy (energy security, energy market) or cost-of-living crisis (energy bill, energy price) evidences the energy crisis as one of the main shocks that need resilience, both from the individual or household level, and from the national economy (see Appendix 1, Table 7).*

*In consonance with the recurrent use of terminology related to funding applications revealed by the wordlists (Table 3), ‘research’ is the most prominent theme identified for the keywords of the funders’ corpus (see Appendix 1, Table 7)— 41 out of the first 50 ‘terms’ of the FundRC refer to research-related matters, such as types of documentation and individual roles involved in applications. Remarkably, if register-specific jargon is excluded, the most salient theme is ‘climate and environment’ (e.g. net zero, climate change) and, in a much lesser extent, economy (supply chain, national capability), which reflects the themes identified in the governmental discourses*

**8.** The study lacks a discussion on future research directions and limitations. Include a section that addresses the limitations of the current study and suggests areas for future research to provide a pathway for further investigation and to acknowledge the boundaries of the current research.

The Concluding discussion section has now been structured in three subsections: 6.1 Findings, 6.2 Limitations and future research directions (copied below), and 6.3 Recommendations.

*6.2 Limitations and future research directions*

*Findings of this study are restricted to the UK context; populations with different cultural, sociopolitical and economic traditions may show different understandings and applications of the concept. Importantly, the limited number of focus group participants makes it difficult, if not impossible, to extrapolate findings to the general population. Lack of demographic information does not make it possible to examine whether understandings of resilience vary across different social groups—e.g. some focus group participants observed that different age groups show different orientations towards the concept, with elder populations conceptualising resilience in relation to material conditions while young populations understand it in relation to psychological wellbeing and the individual ability to seek support. Follow-up studies on lay understandings of the concept should complement focus groups with other public involvement methods which allow for more efficient data gathering and the possibility to scale-up findings, such as questionnaires including both closed and open questions (see, for example, McClaughlin et al., 2023 or Vilar-Lluch et al., 2024 for mixed-methods studies combing corpus linguistics with representative surveys applied to public health guidance reception).*

*While this study specifically focused on examining uses of resilience in governmental discourses and funding calls to identify possible correspondences between these two domains, restricting the data to these registers has inevitably limited the uses of ‘resilience’ to those that appear in these texts. Examining uses of ‘resilience’ in health communication discourses or news media would provide a more encompassing overview of the different conceptualisations and areas of application of the concept, illustrating how specific disciplines adopt it (e.g. health sciences vis-à-vis politics) and how it is communicated to the public.*
